# Comparison of [^11^C]TZ1964B and [^18^F]MNI659 for PET imaging brain PDE10A in nonhuman primates

**DOI:** 10.1002/prp2.253

**Published:** 2016-08-26

**Authors:** Hui Liu, Hongjun Jin, Xuyi Yue, Junbin Han, Hao Yang, Hubert Flores, Yi Su, David Alagille, Joel S. Perlmutter, Gilles Tamagnan, Zhude Tu

**Affiliations:** ^1^ Department of RadiologyWashington University School of MedicineSt. LouisMissouri; ^2^ Department of NeurologyWashington University School of MedicineSt. LouisMissouri; ^3^ Molecular NeuroImagingLLCNew HavenConnecticut; ^4^ Department of NeurosciencePhysical Therapy and Occupational TherapyWashington University School of MedicineSt. LouisMissouri

**Keywords:** Comparison, nonhuman primates, PDE10A, PET radiotracer, tracer kinetics, tracer metabolism, tracer uptake

## Abstract

Phosphodiesterase 10A (PDE10A) inhibitors show therapeutic effects for diseases with striatal pathology. PET radiotracers have been developed to quantify in vivo PDE10A levels and target engagement for therapeutic interventions. The aim of this study was to compare two potent and selective PDE10A radiotracers, [^11^C]TZ1964B and [^18^F]MNI659 in the nonhuman primate (NHP) brain. Double scans in the same cynomolgus monkey on the same day were performed after injection of [^11^C]TZ1964B and [^18^F]MNI659. Specific uptake was determined in two ways: nondisplaceable binding potential (BP_ND_) was calculated using cerebellum as the reference region and the PDE‐10A enriched striatum as the target region of interest (ROI); the area under the time–activity curve (AUC) for the striatum to cerebellum ratio was also calculated. High‐performance liquid chromatography (HPLC) analysis of solvent‐extracted NHP plasma identified the percentage of intact tracer versus radiolabeled metabolites samples post injection of each radiotracer. Both radiotracers showed high specific accumulation in NHP striatum. [^11^C]TZ1964B has higher striatal retention and lower specific striatal uptake than [^18^F]MNI659. The BP_ND_ estimates of [^11^C]TZ1964B were 3.72 by Logan Reference model (LoganREF) and 4.39 by simplified reference tissue model (SRTM); the BP_ND_ estimates for [^18^F]MNI659 were 5.08 (LoganREF) and 5.33 (SRTM). AUC ratios were 5.87 for [^11^C]TZ1964B and 7.60 for [^18^F]MNI659. Based on BP_ND_ values in NHP striatum, coefficients of variation were ~10% for [^11^C]TZ1964B and ~30% for [^18^F]MNI659. Moreover, the metabolism study showed the percentage of parent compounds were ~70% for [^11^C]TZ1964B and ~50% for [^18^F]MNI659 60 min post injection. These data indicate that either [^11^C]TZ1964B or [^18^F]MNI659 could serve as suitable PDE10A PET radiotracers with distinguishing features for particular clinical application.

**Abbreviations**
[^11^C]TZ1964B: 3‐[^11^C]methoxy‐2‐((4‐(1‐methyl‐4‐(pyridin‐4‐yl)‐1H‐pyrazol‐3‐yl)phenoxy)methyl)‐quinoline
[^18^F]MNI659: 2‐(2‐(3‐(4‐(2‐[^18^F]fluoroethoxy)phenyl)‐7‐methyl‐4‐oxo‐3,4‐dihydroquinazolin‐2‐yl)ethyl)‐4‐isopropoxyisoindoline‐1,3‐dione
AIR: Automated Image Registration
AUC: area under the time‐activity curve
BP_ND_: binding potential (nondisplaceable)
CNS: central nervous system
CV: coefficient of variation
DARPP‐32: dopamine and CAMP‐regulated neuronal phosphoprotein 32
DMSO: methyl sulfoxide
DVR: distribution volume ratio
FWHM: full width at half maximum
HPLC: high‐performance liquid chromatography
LoganREF: Logan Reference model
MP‐RAGE: magnetization‐prepared rapid gradient echo
NHP: nonhuman primate
p.i.: post injection
PDE10A: phosphodiesterase 10A
PET: positron emission tomography
PKA: protein kinase A
ROI: region of interest
SRTM: simplified reference tissue model
SUV: standardized uptake value

## Introduction

Phosphodiesterase 10A (PDE10A) is a dual substrate enzyme that hydrolyses cyclic adenosine monophosphate (cAMP) and cyclic guanosine monophosphate (cGMP); its distribution is exclusively within striatal medium spiny neurons which are enriched in the striatum (Fujishige et al. 1999; Coskran et al. 2006). PDE10A regulates cAMP/cGMP downstream signaling cascades (e.g., cAMP/protein kinase A(PKA)/dopamine and CAMP‐regulated neuronal phosphoprotein 32 (DARPP‐32)) that influence various neural functions including ion conductance and synaptic plasticity and plays a key role in regulating dopaminergic signaling in both direct and indirect striatal pathways (Nishi et al. 2008; Girault 2012). Consequently, enhancement of cAMP signaling through PDE10A knock‐out or PDE10A inhibition has been shown to affect locomotor activity and acquisition of conditioned avoidance in rodent studies (Siuciak et al. 2006; Hebb et al. 2008; Schmidt et al. 2008). Moreover, decreased PDE10A expression has been observed both in animal models and in patients with striatal pathologies, including Huntington disease (HD) and Parkinson disease (PD) (Hebb et al. 2004; Giorgi et al. 2011; Russell et al. 2014; Niccolini et al. 2015). These observations provided the rationale for exploring PDE10A inhibition as a therapy for neuropsychiatric and neurodegenerative diseases. Following promising therapeutic advances, tremendous efforts have been put into the development of a suitable radioligand for PDE10A positron emission tomography (PET) imaging. A potent and specific PDE10A PET radiotracer would allow in vivo quantification of PDE10A levels in the brain in a diverse array of central nervous system (CNS) diseases, and also could provide a metric for target engagement during therapeutic interventions. Several PDE10A radioligands have been recently reported for preclinical or clinical studies (Barret et al. 2014; Fan et al. 2014; Kehler et al. 2014; Plisson et al. 2014; Russell et al. 2014; Hostetler et al. 2015; Li et al. 2015; Liu et al. 2015; Niccolini et al. 2015). Although each ligand has its own set of advantages and disadvantages, a close comparison of these PDE10A radioligands could provide detailed information for choosing the appropriate radiotracer for particular clinical application.

**Figure 1:**
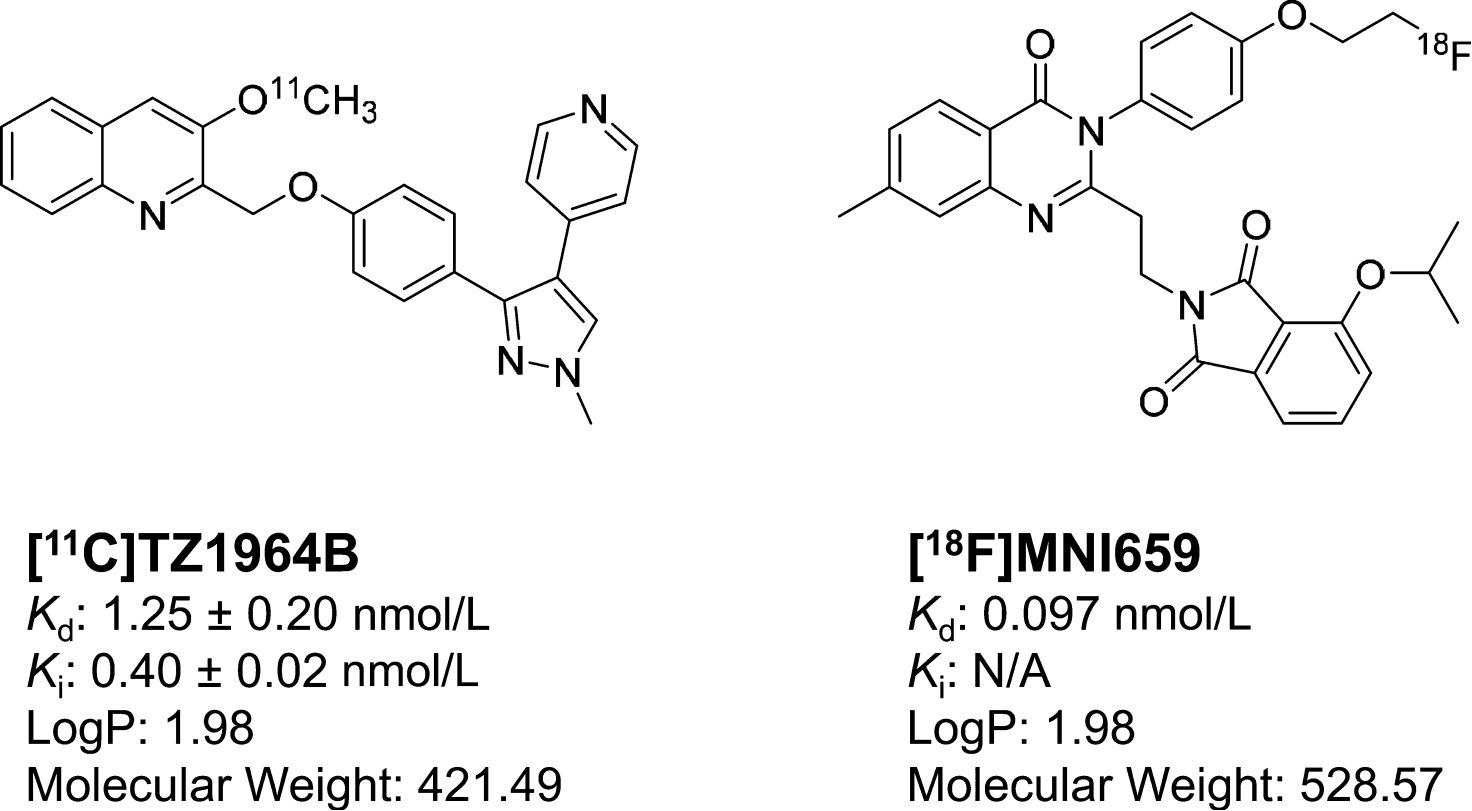
Chemical structures of [^11^C]TZ1964B and [^18^F]MNI659.

In this paper, we have focused on a comparison of two promising radioligands for PDE10A, [^11^C]TZ1964B and [^18^F]MNI659, shown in Figure 1. TZ1964B shows high binding potency and good selectivity toward PDE10A, with a IC_50_ value of 0.40 ± 0.02 nmol/L (Li et al. 2013a). Rat biodistribution and autoradiography studies revealed that [^11^C]TZ1964B had high accumulation in the striatal region and rapid clearance from non‐target brain regions (Fan et al. 2014; Liu et al. 2015), which was further confirmed by microPET studies in NHPs (Fan et al. 2014; Liu et al. 2015). In comparison, MNI659 has subnanomolar binding potency for PDE10A (0.097 nM) (Barret et al. 2014). The reported regional brain uptake of [^18^F]MNI659 in NHPs is consistent with PDE10A distribution: highest in striatal regions and lowest in cerebellum (Barret et al. 2012). [^18^F]MNI659 has also been evaluated for human brain imaging (Barret et al. 2014). TZ1964B and MNI659 have similar lipophilicity and potent in vitro binding affinity for PDE10A, but the radiotracers differ in terms of in vivo kinetics. This study provides a head‐to‐head comparison of [^11^C]TZ1964B and [^18^F]MNI659 for in vivo imaging of the NHP brain by collecting double scans in a same subject in the same day.

## Materials and Methods

### Radioligand preparation

**Figure 2:**
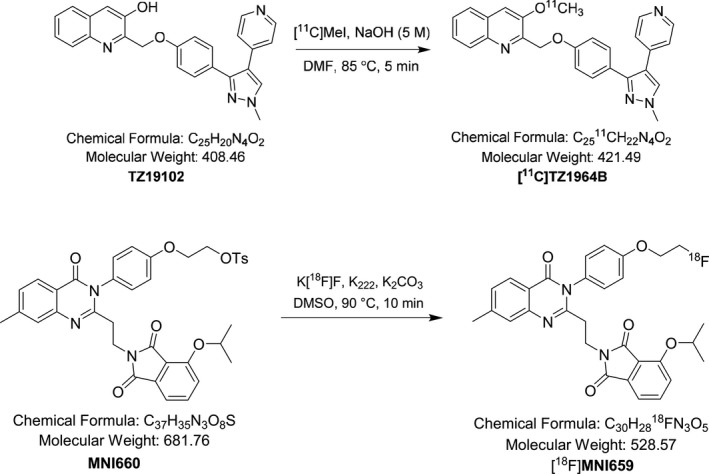
Radiosynthesis Schemes of [^11^C]TZ1964B and [^18^F]MNI659.

Radiosynthesis of [^11^C]TZ1964B. The synthesis of the cold standard and precursor and radiosynthesis of [^11^C]TZ1964B were accomplished following our published procedures (Fig. 2) (Li et al. 2013a; Fan et al. 2014).

Radiosynthesis of [^18^F]MNI659. The radiosynthesis was accomplished following published procedure with modification (Fig. 2) (Barret et al. 2014). Briefly, [^18^F]fluoride was produced in our institution by ^18^O(p, n)^18^F reaction through proton irradiation of enriched ^18^O water (95%) using a RDS111 cyclotron (Siemens/CTI Molecular Imaging, Knoxville, TN). A bolus of aqueous [^18^F]fluoride (~ 200 mCi) was added to a reaction vessel (13 mm × 8 mm) containing Kryptofix 222 (6–8 mg) and aqueous potassium carbonate solution (12.5 *μ*L, 20 mg/mL). The syringe was rinsed with ethanol twice (2 × 0.4 mL). The resulting solution was evaporated under gentle nitrogen flow in an oil bath that was preheated to 90°C. Acetonitrile (3 × 1 mL) was added to the residue and trace amounts of water was azeotropically removed by evaporation. A solution of the tosylate precursor, 2‐(4‐(2‐(2‐(4‐isopropoxy‐1,3‐dioxoisoindolin‐2‐yl)ethyl)‐7‐methyl‐4‐oxoquinazolin‐3(4*H*)‐yl)phenoxy)ethyl 4‐methyl‐benzenesulfonate (3–4 mg, MNI660, provided by Molecular Neuroimaging. Inc, New Haven, CT) in DMSO (250 *μ*L) was added to the ^18^F‐containing reaction vessel. The reaction vessel was capped and swirled with a vortex, and then heated in an oil bath at 90°C for 10 min. The reaction mixture was diluted with 3.0 mL high‐performance liquid chromatography (HPLC) mobile phase (acetonitrile/0.1 mol/L ammonium formate buffer, pH 4.5, 60/40, v/v) and passed through an alumina Neutral Sep‐Pak Plus cartridge, and then loaded onto a HPLC system, which consisted of an Agilent SB‐C18 semi‐preparative HPLC column (250 mm × 9.6 mm) and UV detection at 254 nm, with a flow rate of 4 mL/min. Under these conditions, the retention time of [^18^F]MNI659 was 16–18 min and the retention time of the precursor was 23–24 min. The product [^18^F]MNI659 was collected into a 100 mL vial containing 50 mL sterile water and then passed through a C‐18 Sep‐Pak Plus cartridge. [^18^F]MNI659 was trapped on the Sep‐Pak, then rinsed with 20 mL sterile water, the product [^18^F]MIN659 was eluted into a sterile dose vial using USP grade 10% of ethanol saline solution (6.0 mL). The final dose was delivered for animal studies and quality control (QC) analysis. An aliquot (100 *μ*L) was authenticated by co‐injection with nonradiolabeled standard reference MIN659 on an analytical HPLC system (Agilent SB‐C18, 250 × 4.6 mm, 5 *μ*) with a mobile phase of acetonitrile/0.1 mol/L ammonium formate buffer (75/25, v/v, pH~4.5). Under these conditions, the retention time for [^18^F]MNI659 was ~ 4.6 min at a flow rate of 1.2 mL/min. The whole procedure took about 70 min.

### In vivo microPET studies in NHPs

#### Subjects

All animal experiments were conducted in compliance with the Guidelines for the Care and Use of Research Animals under protocols approved by Washington University's Animal Studies Committee. This work was conducted at the Washington University School of Medicine in St. Louis. Animals were maintained in AAALAC‐accredited housing facilities with access to food and water ad libitum, and 12‐h dark and light cycles; all animals were equally engaged with an enriched environment such as watching movies or playing with appropriate toys.

PET imaging studies were carried out on two adult male cynomolgus monkeys, weighing on 7–8 kg. Subject A underwent three sets of double scans, and Subject B underwent one set of double scans. Animals were prepared for microPET studies as previously reported (Liu et al. 2015). About 1.5–2.5% isoflurane inhalation anesthesia was maintained throughout the microPET imaging sessions. A 20‐gage plastic catheter was inserted into a limb vein to permit hydration and injection of the radiotracer. Arterial blood was collected through another 20‐gage plastic catheter which was placed into a femoral artery.

#### PET data acquisition

The imaging studies of [^11^C]TZ1964B and [^18^F]MNI659 in the NHP brain through double scans were done using a MicroPET Focus 220 scanner (Concorde/CTI/Siemens Microsystems, Knoxville, TN). For all the double scans, [^11^C]TZ1964B studies were performed in the morning and [^18^F]MNI659 in the afternoon, with an interval of ~3 h between the injection of each tracer. Prior to each PET acquisition, a 45‐min transmission scan for attenuation correction was performed following a 10 min transmission that was used to check the position of the brain within the scanner. Subsequently, 296–370 MBq of [^11^C]TZ1964B or [^18^F]MNI659 was intravenously injected, and a 120 min dynamic (3 × 1‐min frames, 4 × 2‐min frames, 3 × 3‐min frames, and 20 × 5‐min frames) PET scan was acquired.

#### PET image processing

The PET images were processed according to the previous report (Liu et al. 2015). Briefly, emission scans were corrected and reconstructed using filtered back projection as described previously (Miller et al. 1989). The final reconstructed resolution was 2.00 mm full width at half maximum (FWHM) for all three dimensions (axial) at center of the field of view. The actual field of view is 25.8 cm diameter and a 7.6 cm axial length. The reconstructed PET images was co‐registered with magnetization‐prepared rapid gradient echo (MP‐RAGE) MR images using Automated Image Registration (AIR) (Woods et al. 1993), and superimposed using Analyze 10.0 (AnalyzeDirect, Overland Park, KS) for quality control. For quantitative analyses, three‐dimensional regions of interest (ROIs) for cerebellum (Subject A: 583 voxels; Subject B: 750 voxels, the voxel size is 1.858 mm × 1.858 mm × 0.796 mm = 2.75 *μ*L), striatum (A: 695 voxels; B: 1158 voxels), frontal cortex (A: 263 voxels; B: 225 voxels), midbrain (A: 64 voxels; B: 118 voxels), and hippocampus (A: 68 voxels; B: 64 voxels) were identified on MRI images and then transformed into PET images using the co‐registration transformation matrix. The ROIs for each animal were kept fixed for all subsequent studies once identified on a baseline scan. Time–activity curves (TACs) for each ROI were then obtained from the dynamic PET images. Standardized uptake value (SUV) of each tracer was calculated and standardized to the body weight and the injected dose of radioactivity.

#### Reference‐based tracer kinetic analysis

Regional tracer uptake was quantified using two different reference‐based kinetic methods: Logan Reference (LoganREF) model (Logan et al. 1990, 1996; Innis et al. 2007; Liu et al. 2015) and simplified reference tissue model (SRTM) (Lammertsma and Hume 1996; Gunn et al. 1997; Innis et al. 2007; Liu et al. 2015). According to the LoganREF model, the distribution volume ratio (DVR) of each ROI was calculated using a MATLAB (Mathworks Inc., Natick, MA). The nondisplaceable binding potential (BP_ND_) was then estimated: BP_ND_ = DVR‐1. BP_ND_ and R1, *k*_2_ values were also obtained from the SRTM model (Lammertsma and Hume 1996; Watabe et al. 1996).

We also calculated the area under the time–activity curve (AUC) for the striatal to cerebellar ratio (AUC‐striatum/AUC‐cerebellum); this is a simple model and input function‐independent method to quantify an index of tracer accumulation in the brain (Muller Herde et al. 2015). The 60–120 min data were used for AUC ratio calculation, since the striatum‐to‐cerebellum SUV ratio reaches a plateau at this time point. Ideally, the AUC ratio equals the DVR determined by LoganREF model (Muller Herde et al. 2015). Thus, bias was calculated as (mean AUC ratio ‐ mean DVR)/mean AUC ratio (Choi et al. 2015).

### Metabolite studies

The radiometabolite analysis was performed according to previous reports (Tu et al. 2011, 2015). Arterial blood samples (1.2–1.5 mL) were collected in a heparinized syringe at 2, 5, 15, 30, and 60 min (for [^11^C]TZ1964B) or 5, 15 30, 60, 90, and 120 min (for [^18^F]MNI659) postinjection of each radiotracer. Plasma (400 *μ*L) was collected by centrifugation of the whole blood and mixed with ice‐cold methanol (0.92 mL) to deproteinate and extract the parent compound and radiolabeled metabolites. After centrifugation, the supernatant was mixed with water (1/1, v/v), and loaded onto a HPLC system with an Agilent SB C‐18 analytical HPLC column (250 mm × 4.6 mm, 5 *μ*) and a UV detector with 254 nm wavelength. The mobile phase was acetonitrile/0.1 mol/L ammonium formate buffer pH 4.5, (52/48, v/v) with a flow rate of 1.0 mL/min. The eluted fractions were collected for 16 min at 1 min intervals; the radioactivity of in each fraction was counted in a well counter, and results were corrected for background radiation and physical decay.

### Log P Measurement

Log P was measured according to the previous report (Li et al. 2013b), by adding different volumes of [^11^C]TZ1964B or [^18^F]MNI659 into a mixture of 1‐octanol (3 mL) and 0.1 mol/L phosphate‐buffered saline (PBS, pH 7.4, 3 mL). The mixture was vortexed and then centrifuged. Then, 2 mL organic layer was mixed with 1 mL 1‐octanol and 3 mL PBS buffer. The resulting mixture was vortexed and centrifuged again. Then 1 mL of the organic layer and 1 mL of the aqueous layer were separated and loaded in a gamma counter. The results were corrected for background radiation and physical decay. The partition coefficient Log D7.4, which equals Log P, was calculated as the decimal logarithm the ratio of cpm/mL of 1‐octanol to that of PBS.

### Statistical analysis

BP_ND_, AUC ratio, and other kinetic parameters of radiotracers were expressed as mean ± standard deviation (SD). Pearson's correlation was performed to determine the relationship between BP_ND_ and AUC ratio. The reported data represented the results of all four double scan experiments. All statistical analysis was processed by Graph Prism 5 (GraphPad Software Inc., San Diego, CA).

## Results

### Radiosynthesis

[^11^C]TZ1964B was obtained in high specific activity >296 GBq/?mol (decay corrected to end of bombardment), with radiochemical yield 20–30% (decay corrected to end of bombardment) and radiochemical purity >99%. [^18^F]MNI659 was obtained in high specific activity 37‐74 GBq/μmol (decay corrected to end of bombardment), with radiochemical yield 20–30% (decay corrected to end of bombardment) and radiochemical purity >99%.

### Comparison of regional brain uptakes

**Figure 3:**
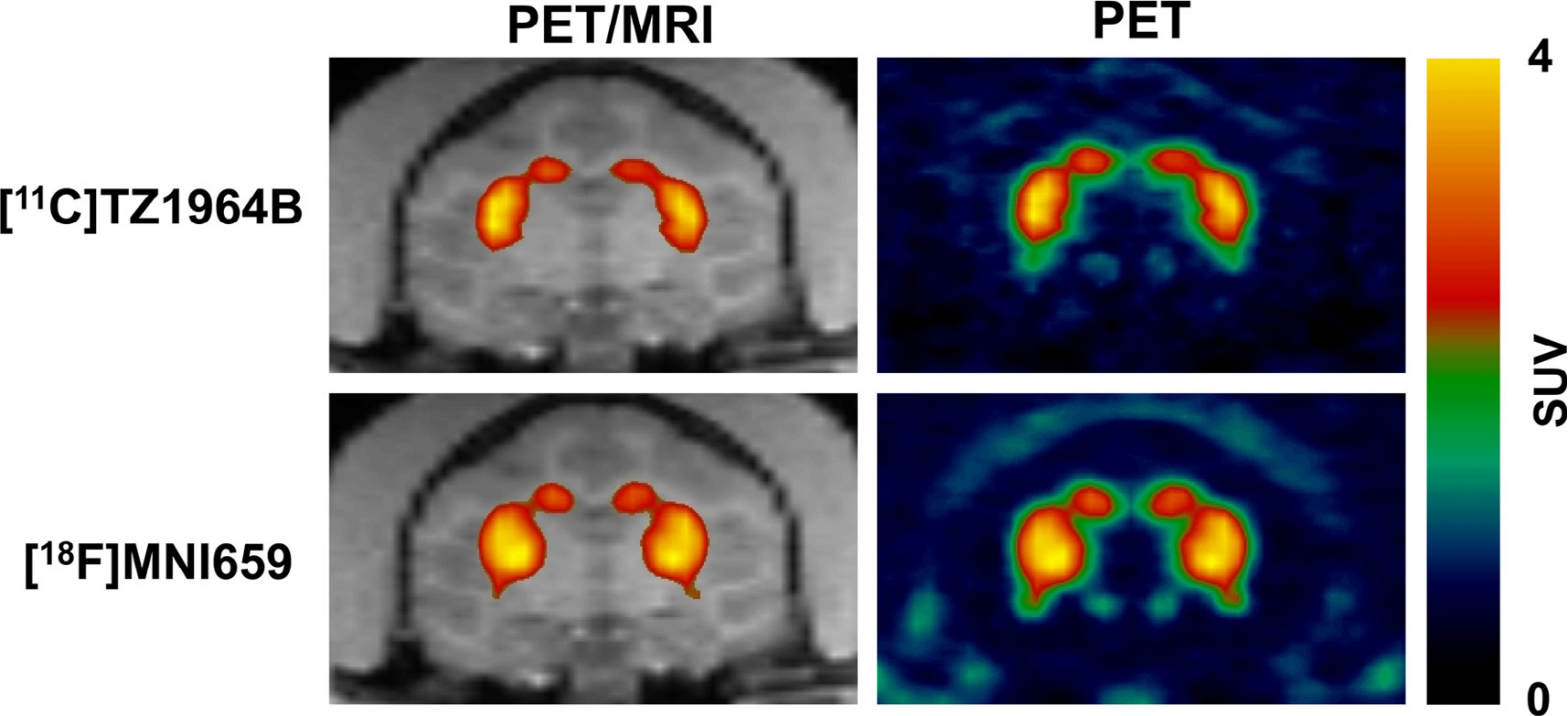
Representative PET images of [^11^C]TZ1964B and [^18^F]MNI659 in NHP brains. Both radiotracers showed high accumulation in NHP striatum (red/yellow areas).

**Figure 4:**
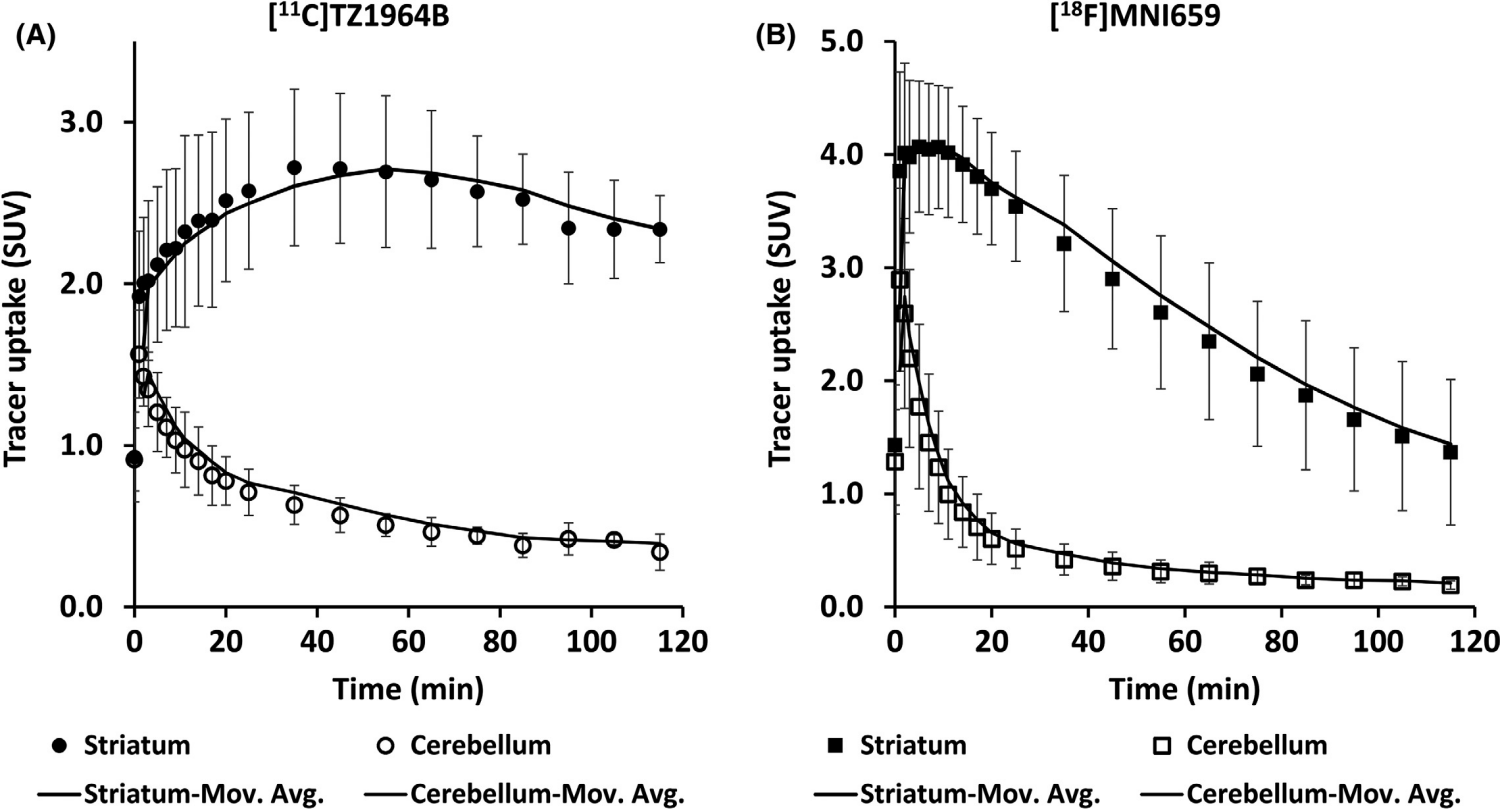
Averaged SUV curves of [^11^C]TZ1964B and [^18^F]MNI659 in the striatum and the cerebellum of NHP brains (*n* = 4 scans in 2 NHPs). Dark lines indicated moving average (Mov. Avg.) trendlines. SUV, standardized uptake value.

Both radiotracers showed high accumulation in NHP striatum (Fig. 3). The regional tracer uptake pattern in the NHP brain was identical for these two radioligands: striatum had highest uptake, followed by midbrain; frontal cortex, hippocampus, and cerebellum, which showed low level of tracer accumulation. (Table 1 and 2). Striatal and cerebellar peak SUV values for [^18^F]MNI659 PET were higher than those of [^11^C]TZ1964B PET (Fig. 4). In [^11^C]TZ1964B PET, striatal uptake reached the max SUV value ~40–60 min p.i. and declined slowly (Fig. 4A). In [^18^F]MNI659 PET, the peak appeared at about 10 min p.i., and declined rapidly (Fig. 4B).

**Table 1:** Binding potential, AUC ratio, and kinetic parameters for [^11^C]TZ1964B and [^18^F]MNI659 in NHP striatum^a^

	[^11^C]TZ1964B	[^18^F]MNI659
AUC ratio (Stri/Cere)	5.87 ± 0.45	7.60 ± 2.68
BP_ND_‐Logan	3.72 ± 0.62	5.08 ± 1.62
BP_ND_‐Logan CV (%)	16.61	31.93
Bias (DVR v.s. AUC ratio)^b^	0.20	0.20
BP_ND_‐SRTM	4.39 ± 0.39	5.33 ± 1.36
BP_ND_‐SRTM CV (%)	8.77	25.5
R1	1.16 ± 0.17	1.20 ± 0.20
k_2_ (min^−1^)	0.13 ± 0.017	0.28 ± 0.13

a *n* = 4 scans in 2 NHPs. Data were presented as mean ± SD.

b DVR = BP_ND_‐Logan + 1. Bias was calculated as (mean AUC ratio ‐ mean DVR)/mean AUC ratio.

**Table 2:** BP_ND_ and AUC ratio values for [^11^C]TZ1964B and [^18^F]MNI659 in other brain regions^a^

	[^11^C]TZ1964B			[^18^F]MNI659		
Regions	AUC ratio	BP_ND_‐Logan	BP_ND_‐SRTM	AUC ratio	BP_ND_‐Logan	BP_ND_‐SRTM
Midbrain	1.51 ± 0.47	0.26 ± 0.20	0.57 ± 0.30	2.59 ± 0.42	1.02 ± 0.14	1.11 ± 0.16
Frontal cortex	1.38 ± 0.13	0.15 ± 0.05	0.43 ± 0.28	1.37 ± 0.12	0.29 ± 0.10	0.29 ± 0.09
Hippocampus	1.35 ± 0.41	0.11 ± 0.25	0.32 ± 0.50	1.08 ± 0.23	0.11 ± 0.18	0.16 ± 0.20

a *n* = 4 scans in 2 NHPs. Data were presented as mean ± SD.

### Comparison of regional binding values and kinetic parameters

**Figure 5:**
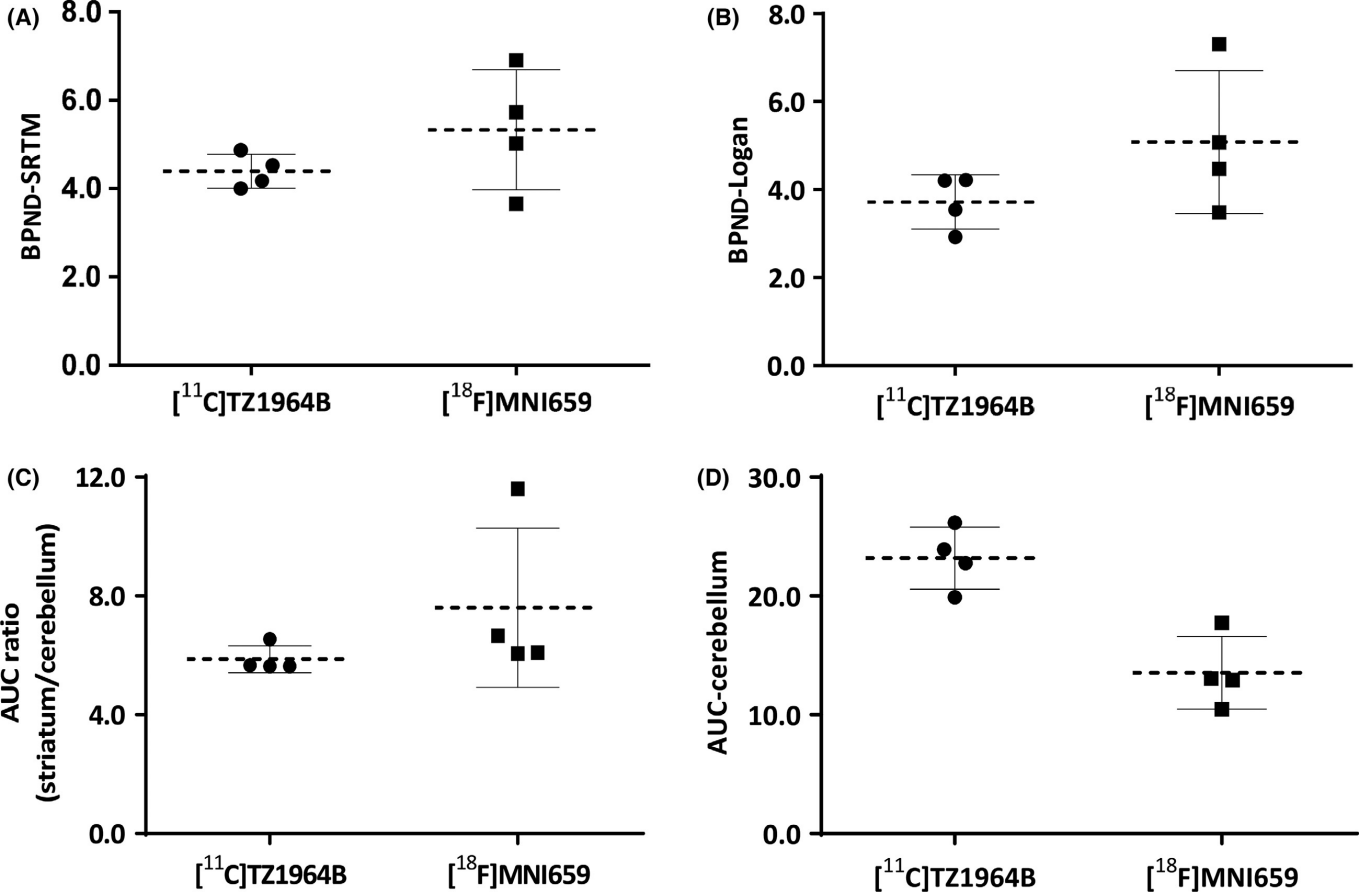
Binding potentials and AUC values (60‐120 min) of striatum and cerebellum of [^11^C]TZ1964B and [^18^F]MNI659 in NHP brains (*n* = 4 scans in 2 NHPs). BP_ND_‐SRTM, binding potential estimated by SRTM modeling; BP_ND_‐Logan, binding potential estimated by Logan modeling; AUC, area under the time–activity curve.

**Figure 6:**
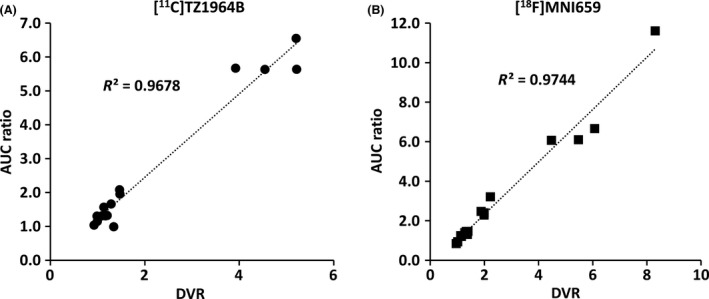
Relationship between the DVR and AUC ratio values for [^11^C]TZ1964B and [^18^F]MNI659 in NHP brains (*n* = 4 scans in 2 NHPs, 4 brain regions included). The AUC ratio values of both tracers strongly correlated with DVR values.

**Figure 7:**
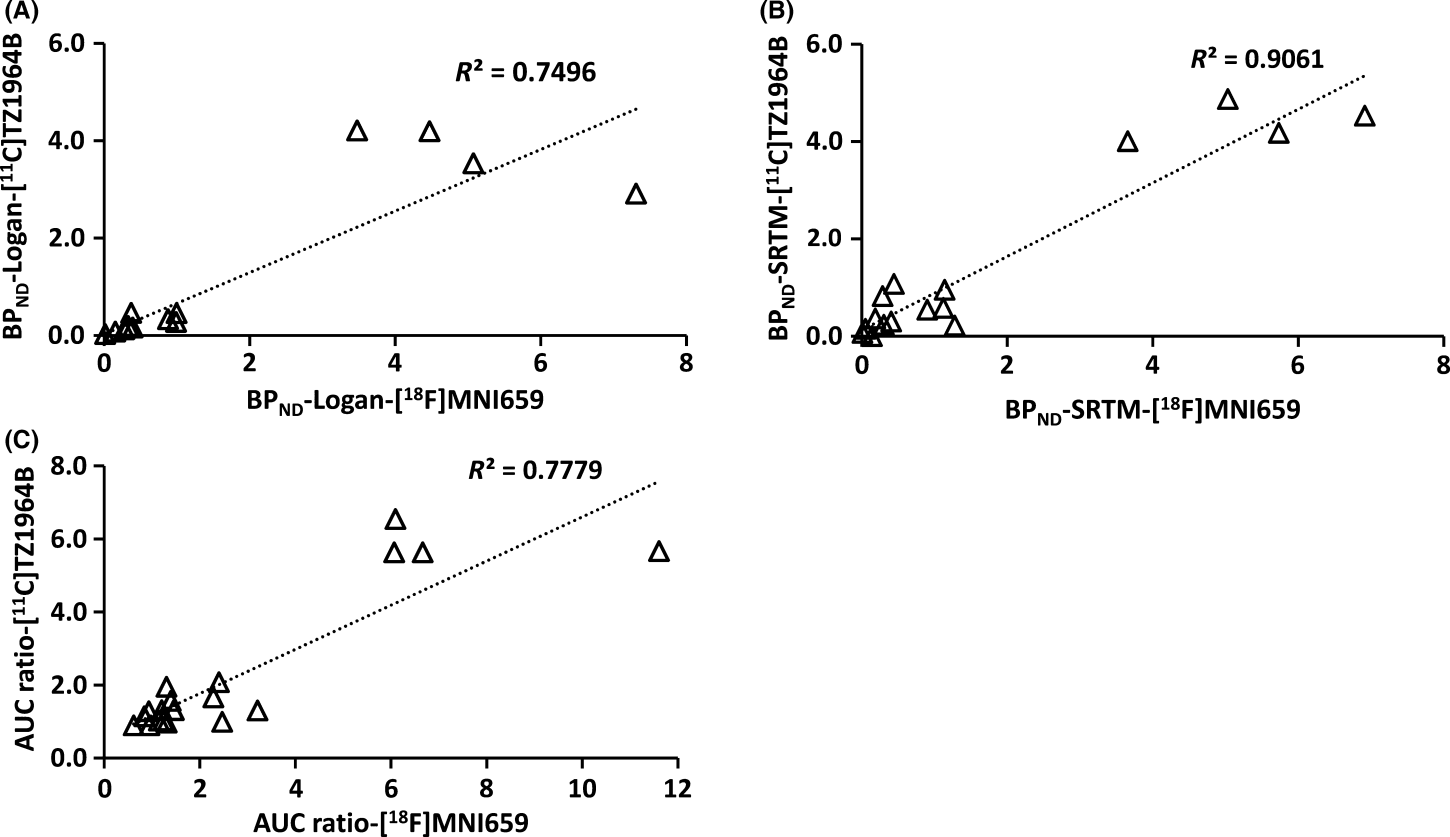
Correlation of BP_ND_ and AUC ratio values between [^11^C]TZ1964B and [^18^F]MNI659 in NHP brains (*n* = 4 scans in 2 NHPs, 4 brain regions included) Strong correlation of BP_ND_ values (either BP_ND_‐Logan or BP_ND_‐SRTM), as well as AUC ratio values, was observed between [^11^C]TZ1964B and [^18^F]MNI659.

BP_ND_, AUC ratio, and kinetic parameters are summarized in Figure**** 5 and Table 1 and 2. The striatal BP_ND_ and AUC ratio values of [^18^F]MNI659 PET were higher than the corresponding values for [^11^C]TZ1964B PET, however, the difference was not statistically significant due to large variation of [^18^F]MNI659 PET values. Notably, [^18^F]MNI659 displayed a higher striatal K_2_ value (twofold) than [^11^C]TZ1964B. For other ROIs, [^18^F]MNI659 had lower cerebellar uptake, determined by AUC, than [^11^C]TZ1964B, and midbrain showed modest uptake of both tracers. The correlations between the AUC ratio values and DVR values were strong and positive for both radiotracers (*R* ^2^ = ~0.97, Fig. 6). However, compared to DVR values, the AUC ratio values were mostly overestimated. Brain regions with high PDE10A‐expression appeared to have higher overestimation than regions with lower PDE10A density. The overestimation of AUC ratio and DVR was similar for both tracers. Strong correlation between BP_ND_ values (either BP_ND_‐Logan or BP_ND_‐SRTM) and AUC ratio values, was also observed for both [^11^C]TZ1964B and [^18^F]MNI659 (Fig. 7).

### Comparison of tracer metabolism and Log P

**Figure 8:**
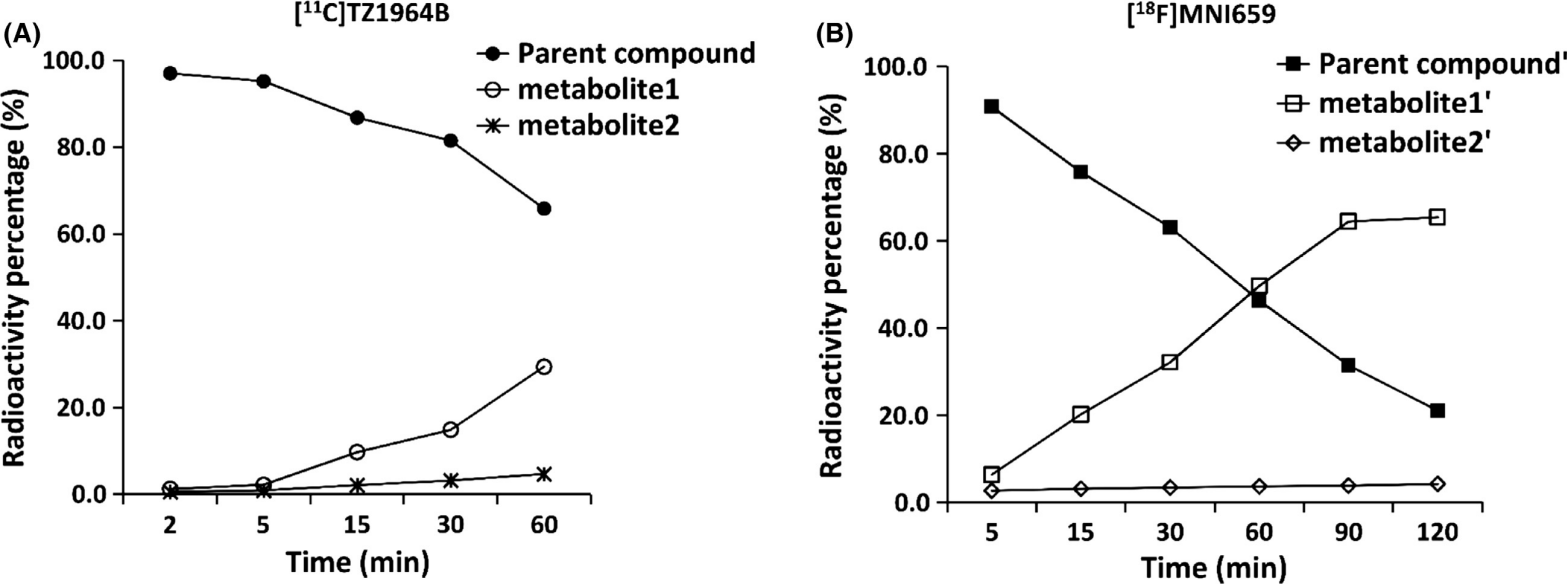
Percent radioactivity in NHP plasma of [^11^C]TZ1964B, [^18^F]MNI659 and the corresponding metabolites (*n* = 1). At 60 min, the percentage of parent compounds were ~70% and ~50%, for [^11^C]TZ1964B and [^18^F]MNI659, respectively.

The fraction of parent radioligand and corresponding radiolabeled metabolites in plasma are shown in Figure**** 8. Analysis of radiolabeled metabolites in plasma extracts of [^11^C]TZ1964B after 60 min p.i. was hampered due to low radioactivity in the plasma at later time points. HPLC radiometabolite analysis revealed [^11^C]TZ1964B was more stable than [^18^F]MNI659 in vivo. At 60 min, the percentage of parent compounds were ~70% and ~50%, for [^11^C]TZ1964B and [^18^F]MNI659, respectively.

The measured Log P values were 1.98 ± 0.09, and 1.98 ± 0.06, for [^11^C]TZ1964B and [^18^F]MNI659, respectively.

## Discussion

In this study, the binding of two potent and selective PDE10A radioligands, [^11^C]TZ1964B and [^18^F]MNI659, was examined and compared in the NHP brain using several quantitative approaches. For an ideal brain PET radiotracer, there are several generally required properties, including small molecular weight, appropriate lipophilicity, high binding affinity, good target selectivity, favorable in vivo kinetics, and metabolism (Fumita and Robert 2002). In this case, molecular weights of both TZ1964B and MNI659 are under 600 Daltons. Moreover, both ligands have similar lipophilicity (measured Log P values equal 1.98 for both ligands), as well as subnanomolar in vitro binding affinity, although the reported binding affinity of MNI659 is higher than that of TZ1964B. According to the crystal structure of PDE10A (Chappie et al. 2012), TZ1964B binds to two key sites in the selectivity pocket, which are the selectivity pocket (S) and the hydrophobic clamp (H). MNI659 and its analogs binds with four key elements of the selectivity pocket, including S, H, the Gln interaction (G), and the exo‐binding site (E); this may result in the observed increased binding affinity to PDE10A compared with TZ1964B.

Although both tracers are able to map PDE10A in the NHP brain, their in vivo binding measures and tracer kinetics are not identical. Using either reference‐based LoganREF method or modeling‐independent AUC ratio method, [^18^F]MNI659 showed higher striatal binding affinity than [^11^C]TZ1964B, which agreed with the in vitro binding data. Due to differences in plasma clearance (fast) and tissue clearance (slow), the apparent DVR values and AUC ratios are larger than the total DVR values and AUC ratios obtained from kinetic modeling, which produces overestimation (Choi et al. 2015). Interestingly, the overestimation of [^11^C]TZ1964B and [^18^F]MNI659 in the striatum are exactly the same (0.20) (Table 1). As a result, this overestimation produces low accuracy of AUC tissue to reference ratio methods hampers its application to BP_ND_ estimates for both [^11^C]TZ1964B and [^18^F]MNI659, regardless of the good correlation between AUC ratio and DVR for both tracers. Notably, the variation of BP_ND_ and AUC ratio data of [^18^F]MNI659 is large, which has also been reported in human brain imaging (Barret et al. 2014). In contrast, [^11^C]TZ1964B has less variance in BP_ND_ calculations in NHP brain, which could translate to improved accuracy if confirmed in humans. In addition, SRTM was also used for the kinetics analysis in this study. The striatal BP_ND_ values calculated by SRTM were higher than LoganREF model, while the resulting striatal CV by SRTM was smaller than LoganREF model. Moreover, the correlation of BP_ND_‐SRTM values between [^11^C]TZ1964B and [^18^F]MNI659 was better than that of BP_ND_‐Logan and AUC ratio. As noted previously (Barret et al. 2014; Liu et al. 2015), SRTM could be an appropriate modeling method for in vivo quantification of PDE10A using either [^11^C]TZ1964B or [^18^F]MNI659.

For in vivo tracer kinetics, [^11^C]TZ1964B had higher striatal retention and relatively slower striatal washout kinetics although the washout from the reference cerebellar region was rapid. Although high potency ligand binding to PDE10A may contribute to slower washout kinetics in some cases (Hirvonen et al. 2010), for this two‐tracer comparison study, there is no change in the PDE10A density of the animal for each double scan. The binding affinity of [^11^C]TZ1964B to PDE10A is comparable to [^18^F]MNI659. These two factors (radioligand binding affinity and target density) would not explain the slower striatal washout kinetics of [^11^C]TZ1964B in this study. One explanation is that the *k*_2_ value, obtained by SRTM modeling, was smaller for [^11^C]TZ1964B than [^18^F]MNI659, thus the efflux rate constant (across the blood–brain barrier) (Liu et al. 2015) for [^11^C]TZ1964B was slower than [^18^F]MNI659. Another possible reason may be increased metabolic stability. [^11^C]TZ1964B was more stable than [^18^F]MNI659 in vivo, which may result in a higher concentration in the brain and higher striatal retention of [^11^C]TZ1964B, compared with [^18^F]MNI659 at the same time point p.i. Nevertheless, the peak striatal uptake time of [^11^C]TZ1964B is 55–60 min p.i. (Fig. 4A) which is acceptable for clinical use. The higher striatal retention suggest more stable binding of [^11^C]TZ1964B to striatal PDE10A, which may improve reproducibility of the PET measurement at later times such as 60 min p.i.

Notably, the pattern of [^11^C]TZ1964B SUV curve in NHP striatum shown in Figure 4A is slightly different from our previous report (Liu et al. 2015). The SUV curve published previously represented one scan from a single animal. This study included data from four scans in two NHPs. Although there was good reproducibility between the two subjects here, between‐subject variation could account for the difference in the shape of the SUV curve from the earlier report. Despite that observation, BP_ND_ estimates by reference modeling methods in the two studies were similar, supporting reliable reproducibility of [^11^C]TZ1964B PET imaging.

In conclusion, despite differences in in vivo tracer characteristics, both [^11^C]TZ1964B and [^18^F]MNI659 could serve as a suitable PET radiotracer for assessment of brain PDE10A level and for measuring target engagement for new therapeutic interventions. Due to the half‐life of radioisotopes, the ^11^C‐labeled tracer could be utilized for repeated or multiple scans in the same subject on the same day, while the ^18^F‐labeled tracer could be used for several sequential PET studies in different subjects and would permit delivery of the radioligand to off‐site imaging facilities within 3 h travel time. [^11^C]TZ1964B has higher striatal retention, so it could provide better measurements for static scan acquisition, for steady‐state analysis methods, or for displacement studies. [^18^F]MNI659 shows higher initial tracer uptake, and could be preferred for short‐term dynamic scans or for measurement of dissociation rates. The results of this comparison study of two PDE10A radiotracers are based on a limited sample size of NHPs, the consistent performance of both tracers gives promise for assessing PDE10A in vivo. Increasing sample size of NHPs and further characterizing these two tracers in human subjects is warranted for selecting the ideal tracer for particular clinical applications.

## Author Contributions

Participated in research design: Liu, Jin, Tamagnan, Tu. Conducted experiments: Liu, Jin, Yue, Han, Yang. Performed data analysis: Liu, Jin, Yue, Flores, Su, and Tu. Wrote or contributed to the writing of manuscript: Liu, Jin, Alagille, Perlmutter, Tamagnan, Tu.

## Disclosures

None declared.
